# Prenatal exposure to Bisphenol-A as a risk factor for infant neurodevelopment

**DOI:** 10.3389/fendo.2025.1645540

**Published:** 2025-08-29

**Authors:** Ivan Hazel Bello-Cortes, Jose Antonio García-García, Manuel Gutiérrez-Aguilar, Daniela Araiza-Olivera, Celia Sánchez-Pérez, Gabriela García-Cerón, Sofia Morán-Ramos, Hugo Tovar, Andrea Bonilla-Brunner, Roeb García-Arrazola

**Affiliations:** ^1^ Department of Food Science and Biotechnology, Faculty of Chemistry, Universidad Nacional Autónoma de México (UNAM), Mexico City, Mexico; ^2^ Department of Education, Hospital General de México, Mexico City, Mexico; ^3^ Department of Biochemistry, Faculty of Chemistry, Universidad Nacional Autónoma de México (UNAM), Mexico City, Mexico; ^4^ Fox Chase Cancer Center, Temple Health, Philadelphia, PA, United States; ^5^ Institute of Applied Sciences and Technology, Universidad Nacional Autónoma de México (UNAM), Mexico City, Mexico; ^6^ Postgraduate Degree in Biological Sciences, Universidad Nacional Autónoma de México (UNAM), Mexico City, Mexico; ^7^ Instituto Nacional de Medicina Genómica (INMEGEN), Mexico City, Mexico; ^8^ R&D Department, Bioplaster Research Inc., Dover, DE, United States

**Keywords:** Bisphenol A, prenatal exposure, neurodevelopment, behavioral assessment, systematic review

## Abstract

It has been established a chronic human exposure to a particular class of chemicals known as endocrine-disrupting compounds (EDCs). Studies conducted *in vitro, in vivo*, and *in silico* have demonstrated that EDCs can disrupt the endocrine system through epigenetic mechanisms. These changes can be heritable and are associated with a wide range of diseases. Since exposure concentrations of these compounds are measured in parts per million (ppm) or even parts per billion (ppb), a critical question arises: does this pose a significant risk to humankind and future generations? We conducted a comprehensive review of human epidemiological data to provide an assessment of the risk of neurodevelopmental disorders in children associated with maternal exposure to Bisphenol A (BPA). BPA is one of the most studied and relevant EDC’s related to food exposure. Our analysis reveals a correlation between BPA exposure during pregnancy and behavioral issues in offspring on 80% of the reviewed articles. Notably, male infants exposed to BPA during the third trimester exhibited a heightened risk. Our findings highlight the importance of considering potential new health regulations aimed at safeguarding the fetal environment and reducing the risk of neurodevelopmental disorders in children.

## 1 Introduction

The globalization and commercialization of the food industry have significantly promoted the widespread adoption of plastic packaging for the purposes of transportation and preservation. For example, extensive use of polycarbonate packages and containers, as well as cans with an inner coating of epoxy resins, have been shown to exude Bisphenol A (BPA) (1). BPA is a monomer used to produce polycarbonate polymers and has the potential to leach into food items upon contact and be ingested by humans (2).

### 1.1 Health effects of Bisphenol A

Exposure to BPA has been consistently linked to a broad spectrum of adverse health outcomes. These include, but are not limited to, reproductive toxicity, manifesting as infertility, menstrual irregularities, and dysfunction of the testicular and ovarian systems (3–5). Metabolic disorders such as obesity, insulin resistance, and type 2 diabetes (3, 6). Furthermore, immunological disturbances, including immunosuppression and chronic inflammation (7, 8), along with neurotoxicity, characterized by altered brain development and behavior (3, 9). Besides, an elevated risk of hormone-dependent cancers has also been observed in relation to BPA exposure (3, 10). Significantly, these detrimental effects can emerge even at low doses and during critical developmental windows, notably gestation and infancy (11).

The understanding of BPA’s long-term effects is further informed by the Developmental Origins of Health and Disease (DOHaD) hypothesis. This paradigm posits that early developmental environmental factors, encompassing nutrition, stress, and toxicant exposure like BPA, possess the capacity to “program” an individual’s susceptibility to chronic diseases and neurobehavioral disorders later in life (12). Current research endeavors are focused on identifying critical windows of susceptibility and elucidating the underlying molecular mechanisms, with a particular emphasis on epigenetic processes (13).

### 1.2 BPA exposure, metabolism, and regulatory landscape

Most daily human exposure to BPA comes from its presence in food and beverages (14). For instance, the World Health Organization & Food and Agriculture Organization of the United Nations reported in 2011 that canned food for adults contains BPA concentrations from 10 to 70 μg/l and canned beverages from 10 to 23 μg/l (15). The highest concentrations of BPA levels were found in vegetables (0.149 μg/g in green beans) followed by meat (0.0057 μg/g in chicken breast) (14). A study by the Food and Drug Administration (FDA) estimated an average daily intake of 0.2 μg/kg-bw for a population 2 years and older in the United States, with 90% of the population consuming up to 0.5 μg/kg-bw (16).

**Figure 1:**
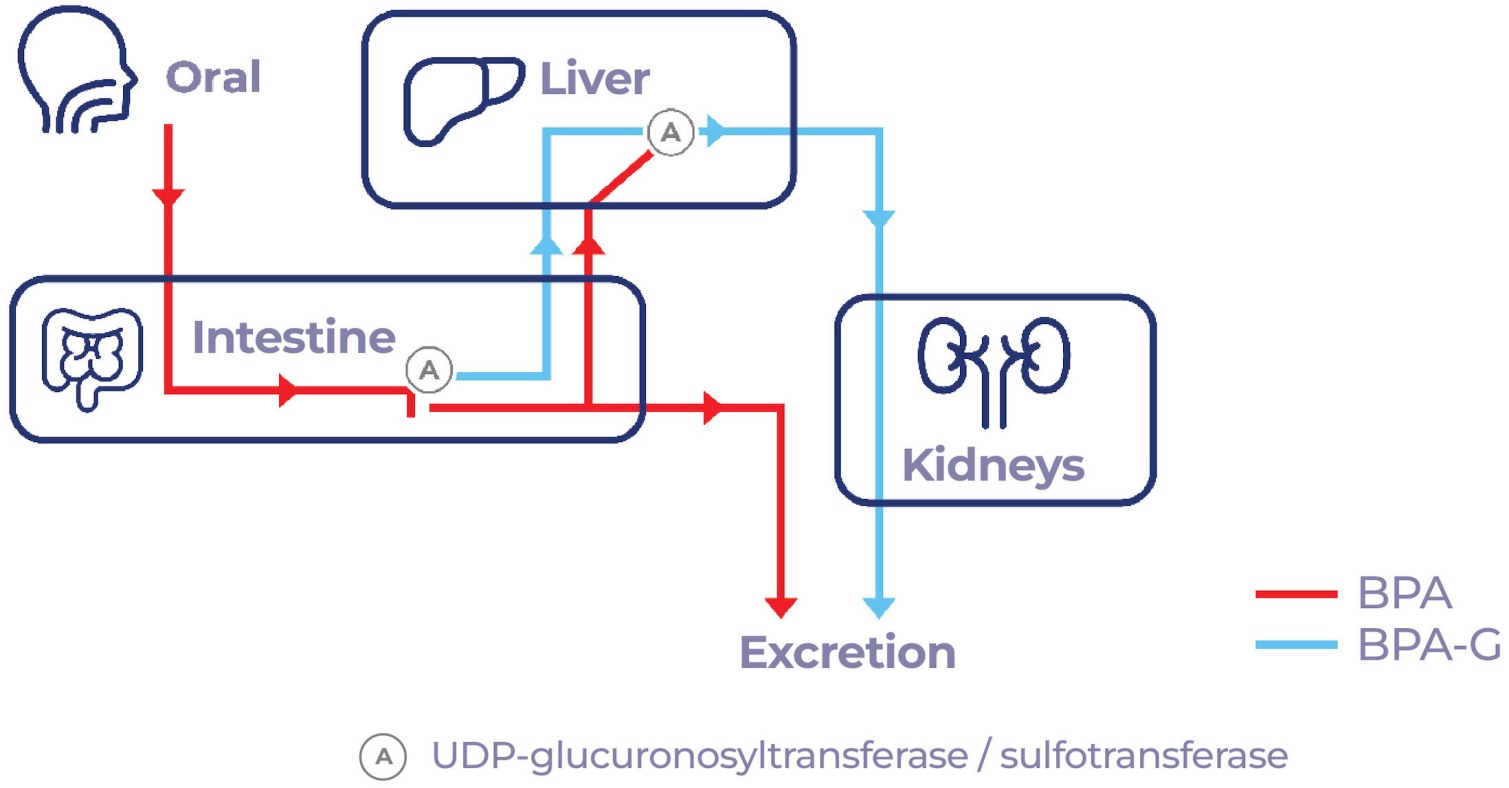
BPA metabolism. BPA, introduced into the body orally, is rapidly metabolized in the intestines and liver, converting it into its inactive state, BPA glucuronide.

**Figure 2:**
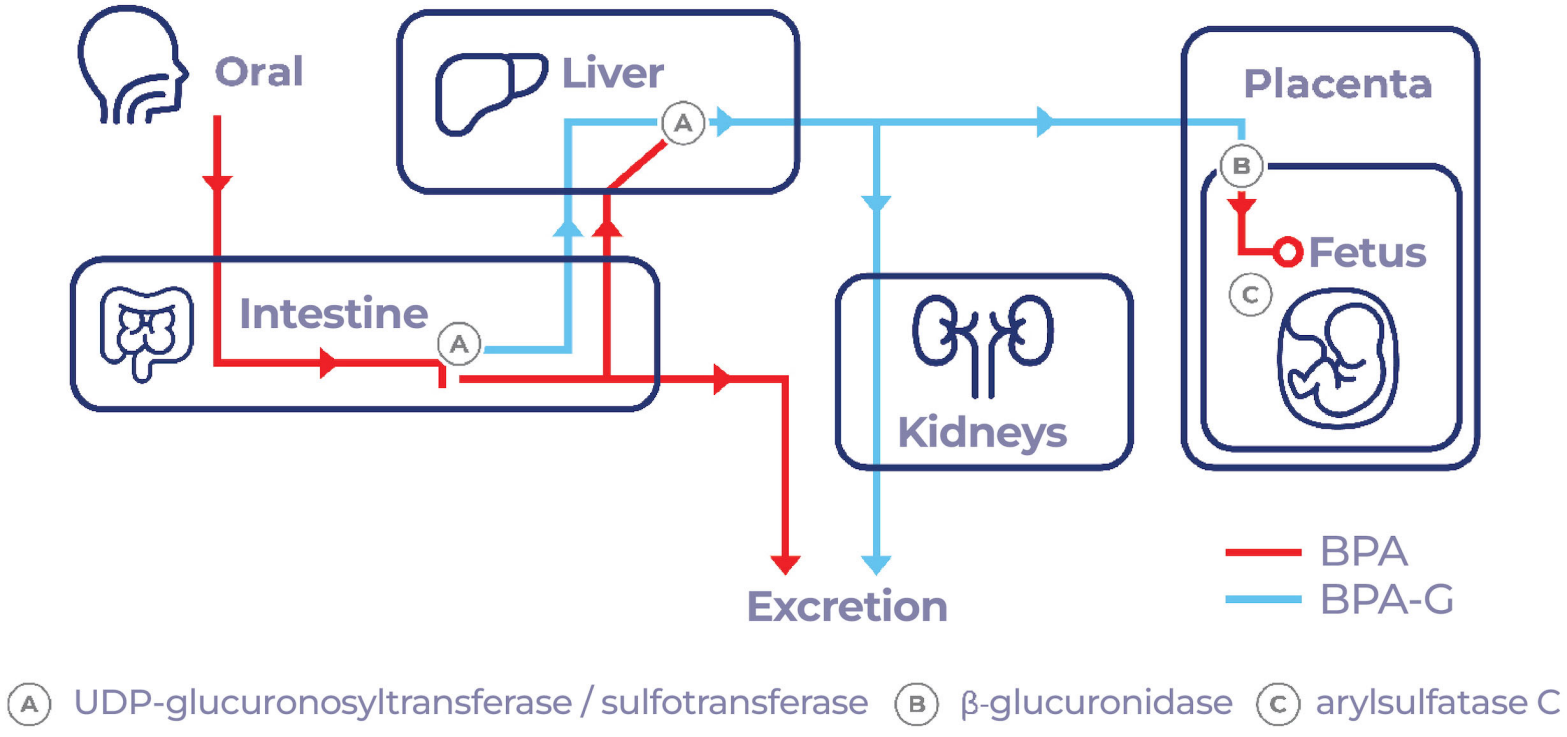
BPA metabolism during pregnancy, BPA is activated again in the placenta to free BPA by means of the enzyme β-glucuronidase. The fetus could inactivate BPA by sulfation, but not by glucuronidation; however, arylsulfatase C activates it again.

**Figure 3:**
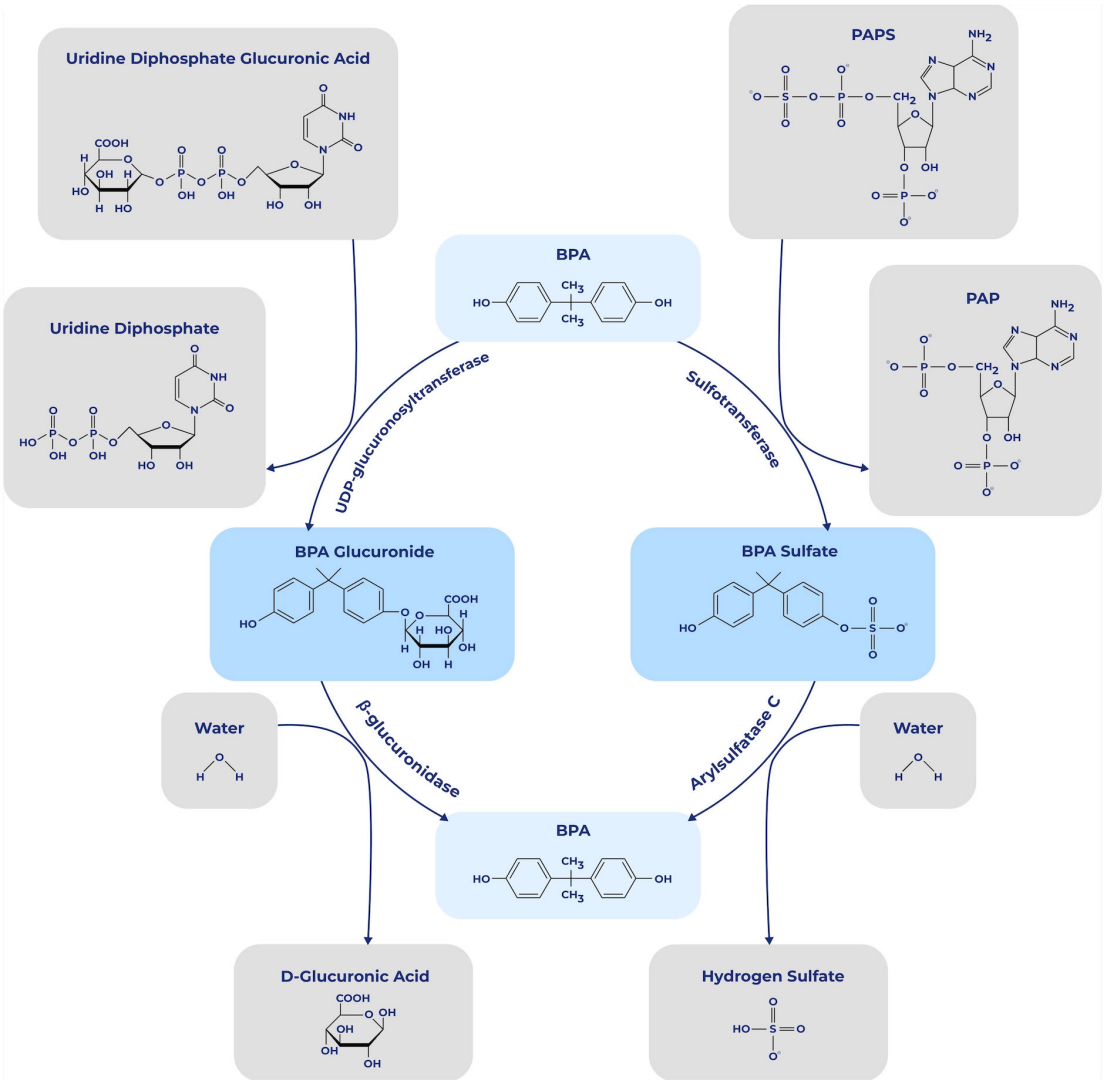
Activation and metabolic deactivation of BPA. Process of deactivation of BPA through glucuronidation and sulfation, and its reactivation through β-glucuronidase and Arylsulfatase C. 3′-phosphoadenosine-5′-phosphosulfate (PAPS), 3′-phosphoadenosine-5′-phosphate (PAP).

Once BPA enters the body along with food, it is absorbed by the gastrointestinal system and transported to the liver to be rapidly metabolized into BPA glucuronide and, to a very small proportion, into BPA sulfate (17). BPA is almost completely bio-transformed by glucuronidation (99.9%) before reaching the bloodstream and subsequently eliminated via urine (18) (**Figure 1**). However, BPA does not remain in its conjugated form in certain tissues, including the placenta in pregnant women (**Figure 2**), where glucuronidases can deconjugate BPA and return it to its free form, leading to a likely fetal exposure (19). According to Bolognesi et al. (20), the fetus cannot eliminate free BPA through glucuronidation due to its low metabolic maturity. There is also evidence that sulfation of BPA may be transformed to its free state by arylsulfatase C, which is found in estrogen responsive (ER) tissues (19) (**Figure 3**). While traditionally BPA metabolites were considered inactive, recent studies indicate that conjugated metabolites can indeed alter cellular functions, including the modulation of energy metabolism and immune responses in neutrophils and urothelial cells (21). Active metabolites such as 4-Methyl-2,4-bis(4-hydroxyphenyl)pent-1-ene (MBP) has been demonstrated to act as a potent estrogenic agonist, capable of promoting breast cancer cell proliferation and disrupting cardiovascular function (22). Glucuronidated and sulfated metabolites may additionally impact adipogenesis, favoring white fat accumulation and impeding brown fat formation (23). The regulatory landscape surrounding BPA focuses on establishing safe exposure doses for humans. The No Observed Adverse Effect Level (NOAEL) is defined as the dose at which no detectable adverse effects are observed. Adverse effects may manifest as biochemical, anatomical changes, or functional failures within the organism under defined experimental conditions. The Tolerable Daily Intake (TDI) is an empirically derived value obtained by dividing the NOAEL by a protection factor for humans. This factor accounts for the possibility that humans may exhibit a ten-fold greater susceptibility to a toxic effect than animals, owing to increased sensitivity, enhanced bioactivation, and a slower elimination rate in humans (24). In the United States, the equivalent of the TDI is termed the Reference Dose (RfD), which is determined through a similar methodology (25).

In 2008 the FDA generated its first draft risk assessment and established a NOAEL of 5,000 μg/kg bw/day for systemic toxicity (26), a limit confirmed in 2009, 2011 (27) and in 2014 (16). Although the United States Environmental Protection Agency (EPA) established in 1988 a Reference Dose (RfD) of 50µg/kg bw/day based on a reduced body weight in a Rat Chronic Oral Bioassay (28), the FDA did not establish a formal acceptable daily intake (ADI) since BPA monomers are not an additive and dictated that the intake in humans is over 27,000 times lower than lethal doses in animals (26).

BPA has garnered attention as an endocrine disrupting compound (EDC) from various food safety institutions globally which, in turn, have conducted diverse risk analyses regarding BPA ingestion within their populations. Countries like Canada and Australia have stated that the levels of BPA consumption among their residents do not pose a health risk. Nevertheless, in 2010, both nations discontinued the use of this material in baby bottles (29, 30). In 2012, the United States banned the material from baby bottles (31) and a year later it made an amendment withdrawing the use of epoxy resins from cans of infant milk formula (32). In the European Union, the use of BPA in food contact materials was allowed in 2011 (33) and banned in baby bottles and training cups (34). In 2018, the European Union amended its regulations, reducing the specific migration limit for polycarbonate and epoxy resins in food contact materials from 0.6 to 0.05mg/kg and extending this standard to encompass varnishes and coatings used in contact with food (35). In 2023, EFSA pointed out a temporary tolerable daily intake (t-TDI) of 0.2ng/kg bw/day (36).

### 1.3 Mechanisms of BPA toxicity

Exogenous compounds that interfere with the synthesis, secretion, and elimination of hormones responsible for reproduction, development, homeostasis, and behavior are collectively termed endocrine disruptors (EDs) (37). Disruption mechanisms can occur via both genomic and non-genomic pathways. The genomic pathway involves EDs binding to hormonal receptors of gene promoters and target cells, leading to conformational changes in protein production and influencing the function and regulation of gene expression. Conversely, the non-genomic pathway manifests when EDs bind to plasma membrane hormone receptors, triggering signal cascades that activate protein second messengers, ultimately leading to alterations in hormonal signaling and synthesis (38). Therefore, EDs perturb the endocrine system, which in turn alters the synthesis of regulatory hormones within the human body, resulting in endocrine-related cancers, reproductive problems, and neurodevelopmental alterations. These alterations may manifest even decades following exposure to EDs (3, 39).

**Figure 4:**
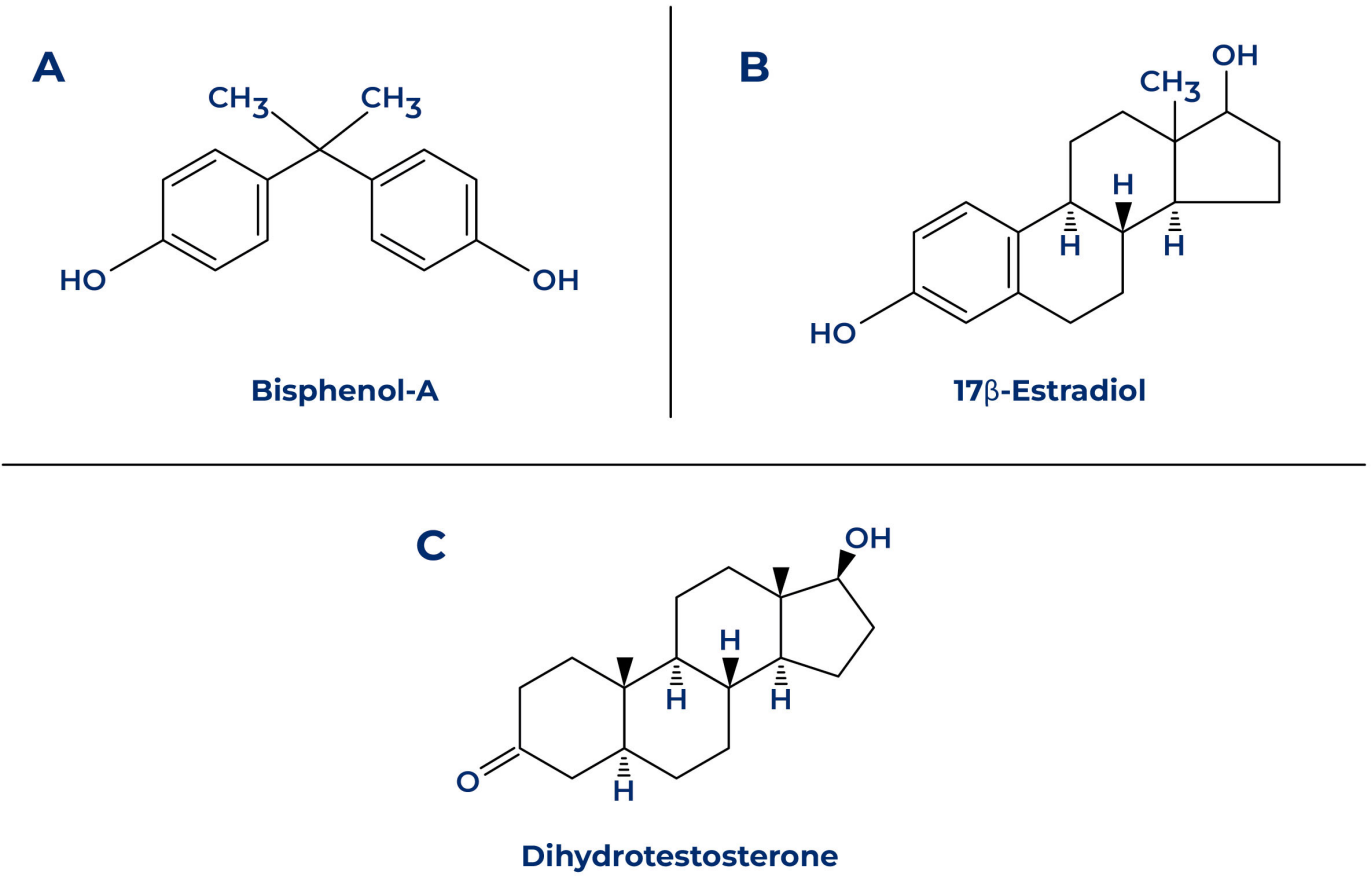
Structural features of bisphenol A, estradiol-17β and dihydrotestosterone, modified from (40).

Unconjugated BPA induces its deleterious effects by binding estrogen and antagonizing androgen receptors, making them unresponsive to its natural ligand, the hormone estradiol-17β and dihydrotestosterone (**Figure 4**). Consequently, BPA is classified as an endocrine disruptor or a xenoestrogen as it prevents proper binding of the hormone to its receptors (41). In more detail, Bisphenol A acts as a ligand for nuclear ER receptors ERα and ERβ, which are found in the cell nucleus with a lower affinity for BPA than for estradiol. Nevertheless, BPA has an 80-fold higher affinity for the ERRγ receptor, which is found in the nucleus of placental and brain cells (42, 43). The receptor-ligand complex is characterized by its dynamic nature, allowing for dissociation and reversibility (44). BPA can also bind, as an agonist, the GPR30 receptor on the cell membrane and the hormone-binding globulin, leading to rapid activation of downstream signaling pathways contributing to testosterone-estrogen imbalances and interfering with neuroendocrine regulation; however, these effects are transient and contingent upon the sustained presence of BPA (42, 43, 45).

Exposure to low doses of BPA, even below established safety limits, has been shown to cause significant adverse, some studies even suggest a non-monotonic dose-response curve, where lower exposure levels may be associated with different ailments (46). The existence of a non-monotonic dose response, where lower exposures may be linked to worse outcomes, challenges traditional toxicological assumptions of a linear dose-response relationship (47). This implies that a simple reduction in BPA levels might not eliminate the risk, and in some cases, chronic very low exposures could be more problematic than acute higher ones.

### 1.4 Neurodevelopmental implications of BPA exposure

Neurodevelopmental deficits can be identified through the symptoms of common mental disorders, which are divided into internalizing and externalizing behaviors. Internalizing behaviors are non-visible attitudes which affect the individual’s mental health, such as depression, anxiety, learning difficulties and lack of attention. Externalizing behaviors are noticeable attitudes in the person, such as hyperactivity, aggressiveness, or defiance of rules (48). Although clinical research in humans is still limited, results are inconsistent as reported in a recent review of the effects of EDCs on child neurodevelopment (49). In different studies, urinary BPA concentrations during pregnancy were associated with externalizing traits (e.g., inattention, hyperactivity) in 2-year-old girls (50), and with lower Intelligence Quotient (IQ) in boys at age 7 (51). In addition, Stacy et al. (52) found that BPA exposure during pregnancy acted as a possible environmental contributor to increased risks of neurobehavioral problems in children. Casas et al. (53) reported an increase in externalizing and internalizing (e.g., depression and anxiety) traits in children upon exposure to BPA during gestation, where mothers presented urine concentrations around 2.29 μg/l. Consequently, the mean value reported by Casas et al. (53) of the estimated prenatal exposure of BPA associated with neurodevelopment in children is 0.057 μg/kg bw/day. This value is 3.5 times lower than the exposure estimated by FDA in 2014.

This compelling set of evidence asserts the need to propose a new risk analysis by the corresponding health authorities. There is already a precedent in 2015 where EFSA determined to reduce the TDI from 50 to 4μg/kg bw (54) and then from 4μg/kg-bw to 0.2ng/kg-bw in 2023 (36). Moreover, we acknowledge the FDA’s perspective that average BPA exposure values estimated probabilistically, in conjunction with international policies aiming for a global reduction in BPA use in food containers, are likely to result in reduced risks to the population. Nevertheless, it’s crucial to note that these assumptions are rooted in BPA exposure beginning at 2 years of age. In contrast, the evidence presented in this paper primarily focuses on human exposure during pregnancy and its effects on children. Based on the above, the main aim of the present work is to make a comprehensive review of the existing scientific literature in humans in which prenatal exposure to BPA and its association with infant neurodevelopment is studied. To our knowledge, this is the first systematic review focusing solely on humans and the effects of BPA exposure on neurodevelopment during the prenatal period in the mother-child pair.

## 2 Materials and methods

### 2.1 Systematic review

The documentary search was conducted using Science Direct and Pubmed, employing the following keywords: BPA, Bisphenol A, neurodevelopment, prenatal, and endocrine disruptor. Inclusion criteria included articles were in English and published between January 2009 and March 2025.

**Figure 5:**
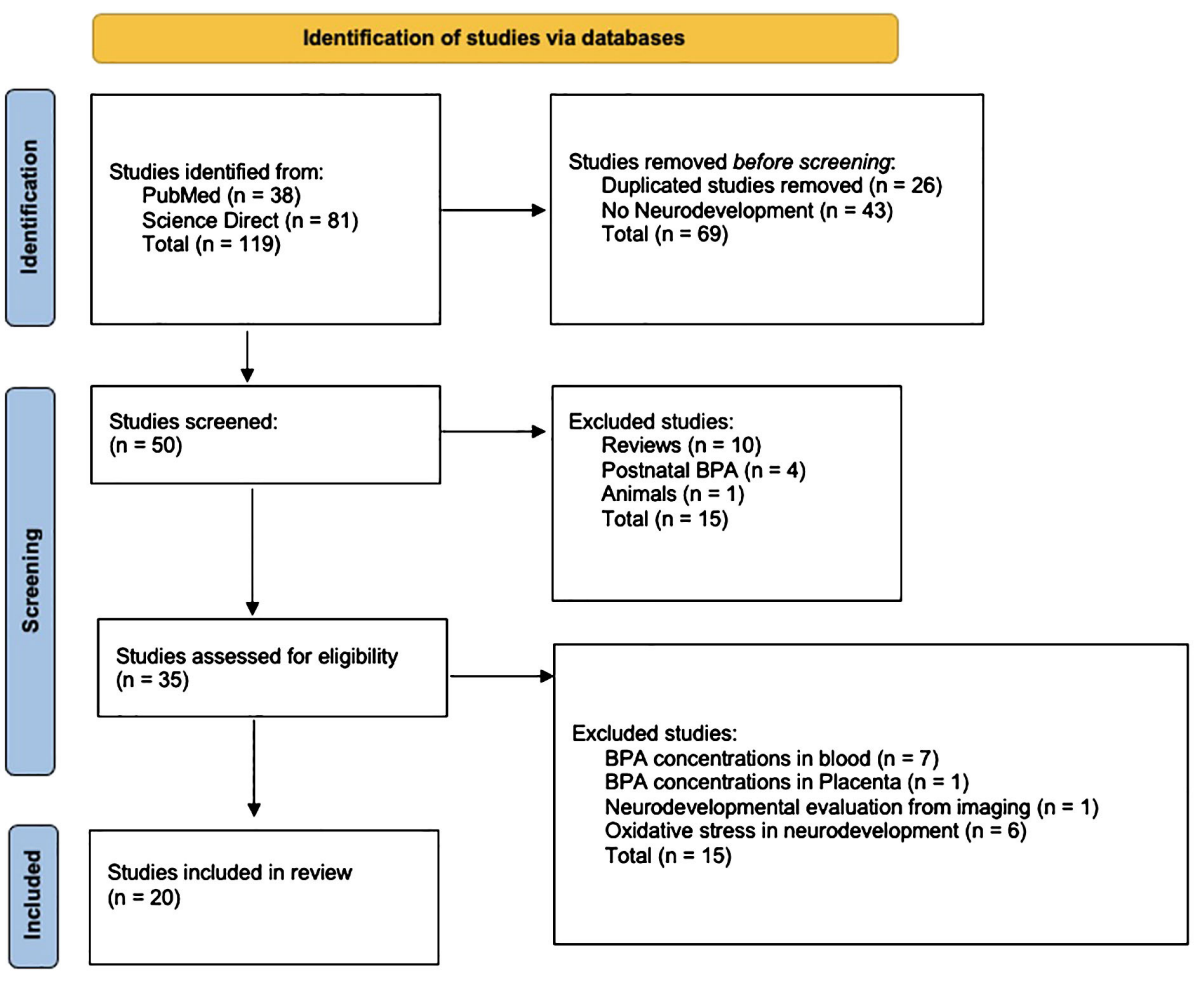
PRISMA flowchart illustrating the study identification process in this review. The diagram depicts the selection of articles from databases, the number of studies excluded, and the screening process for eligibility.

From all the obtained texts, we identified those that specifically examined the impact of prenatal BPA exposure on children’s neurodevelopment where exposure levels were determined through urine samples collected from mothers at various trimesters during gestation (**Figure 5**). The following exclusion parameters were used for that purpose:

- Duplication.
- The evaluation of BPA effect on any other health condition besides neurodevelopment.
- The inclusion of postnatal exposure to BPA.
- The text was a review.
- The text involved animal studies.
- The sample used to obtain BPA concentrations was other than urine from pregnant women.
- The methodology to evaluate neurodevelopment in children was other than observational techniques.

### 2.2 Calculation of daily intake

The calculation of Daily Intake (DI) (µg/kg body weight) of BPA in pregnant women based on urine concentration (UC) was conducted using Equation 1, following the methodology outlined by Huang et al. (55). A urine volume (UV) of 1.5 L averaged over 24 hours and an average weight (W) of 60 kg were employed according to (56).


(1) $$DI=\;\frac{\left(UC\right)\left(UV\right)}{W}$$

Magnitude analysis.

The reviewed studies referred to the BPA effect magnitude on neurodevelopment as the significant difference (β) that existed between the results in population exposed to the different BPA doses included in their paper. β, or the BPA magnitude effect, was obtained as the estimate produced by a multiple linear regression model. As shown in Equation 2, Y is the neurodevelopment test’s result and X_1_ corresponds to the urine BPA concentration. β is in score/dose units, which shows the interaction effect of both variables.


(2) $$Y=\left(\beta_{0}+\beta_{1}X_{1}+\beta_{2}X_{2}+\cdots +\beta_{n}X_{n}\right)+e$$

As different variables were introduced in the model such as BPA concentration, sex and/or age, the authors only took the β value that corresponded to concentration and stablish a 95% confidence interval, where a p<0.05 value points out a significant effect.

To assess the consistency of results across the various tests employed in these studies, a comprehensive analysis was conducted. Utilizing the Review Manager 5.4 software, we generated a forest plot, based on fixed effects model, for both behavioral and cognitive ability tests along with the study weight and heterogeneity across studies. This plot illustrates the effect (β) and their associated confidence intervals. A greater distance of both the effect (β) and the confidence interval from zero indicates increased significance of the result, as the effect (β) signifies the slope of a linear estimate. This visualization allows for a nuanced understanding of the magnitude and significance of the outcomes from each evaluation.

## 3 Results and discussion

### 3.1 Prenatal exposure to Bisphenol A and its association with childhood neurodevelopment

As shown in **Figure 5**, the documentary search led us to initially identify 119 texts, of which 26 were eliminated because of duplication. Subsequently, 43 articles were excluded because, although discussing prenatal exposure, they primarily addressed the effects on conditions such as obesity, respiratory allergies, or cancer.

Following this initial screening, 50 articles remained. From these, 35 were chosen for further analysis. The remaining 15 were excluded as they either constituted reviews of prenatal BPA exposure’s impact on overall human health, involved animal studies, or concentrated on the effects of postnatal exposure on the neurodevelopment of children.

To standardize the data, we excluded studies assessing neurodevelopmental effects based on BPA concentration in placenta or blood (n = 8) and those employing methodologies like imaging or oxidative stress analysis rather than systematic evaluations with observational techniques (n = 7). The final data set consisted of the results of 20 articles (**Table 1**).

**Table 1:** Human clinical studies studying prenatal exposure to Bisphenol A (BPA) as a risk factor in infant neurodevelopment.

#	Studies	Mean [BPA] in urine(μg/L) unadjusted	DI in μg/kg-bw/day	Trimester of pregnancy	Child age (years)	n	Male	Female	Response observed	Effect	Expression of alteration in neurodevelopment	Sex more affected	Test
1	(57)	1.53	0.038	1	7	718	356	362	Skills	Yes	Low IQ	Masculine	WISC-IV
2	(58)	2	0.05	1	2	249	118	131	Behavior	es	Externalizing behavior	Both	BASC-2
3	(59)	1.55	0.039	1	7	803	400	403	Skills	No	NA	Both	WISC-IV
4	(60)	1.1	0.028	2	7	288	133	155	Behavior	Yes	Internalizing behavior	Masculine	BASC-2
5	(61)	1.13	0.028	2	2	456	245	211	Skills	Yes	Reduced mental development.	Both	BSID
6	(62)	1.12	0.028	3	6 to 10	153	77	76	Behavior	Yes	Somatic problems	Masculine	CBCL/DSM
7	(63)	2.96	0.074	3	10	386	206	180	Behavior	Yes	Total difficulties	Masculine	SDQ
8	(64)	1.95	0.049	3	3 to 5	198	87	111	Behavior	Yes	Emotional reactivity and aggressive behavior	Masculine	CBCL
9	(65)	1.9	0.048	3	7 to 9	250	115	135	Behavior	Yes	Externalizing behavior	Masculine	CBCL
10	(65)	1.9	0.048	3	7 to 9	250	115	135	Behavior	Yes	Internalizing behavior	Masculine	CBCL
11	(63)	2.78	0.07	3	7	326	186	140	Skills	Yes	Lower IQ	Masculine	C-WISC
12	(66)	1.2	0.03	3	1.5 to 3	535	282	253	Skills	Yes	Reduced language development	Masculine	MC-CDI
13	(67)	1.95	0.049	3	2	545	254	291	Skills	Yes	Reduced cognitive development	Masculine	BSID III
14	(68)	1.8	0.045	3	2	140	69	71	Skills	Yes	Reduced mental development	Feminine	BSID II
15	(69)	0.8	0.02	3	3	106	55	51	Skills	Yes	Reduced early learning composite	Feminine	MSEL
16	(53)	2.29	0.057	3	1	382	196	186	Skills	Yes	Reduced psychomotor development	Both	BSID
17	(66)	1.2	0.03	3	1.5 to 5	658	348	310	Behavior	No	NA	Both	CBCL
18	(70)	1.2	0.032	3	7 to 9	137			Behavior	No	NA	Both	SRS
19	(71)	0.48	0.012	3	1	368	188	180	Skills	Yes	Reduced neurodevelopment in general, social, and adaptive behavior	Both	GSD
20	(71)	0.48	0.012	3	2	296	152	144	Skills	Yes	Reduced neurodevelopment of language	Both	GSD
21	(72)	2	0.05	All	3	240	112	128	Behavior	Yes	Reduced hyperactivity	Masculine	BASC-2
22	(58)	2	0.05	All	2	249	118	131	Behavior	Yes	Externalizing behavior	Feminine	BASC-2
23	(72)	2	0.05	All	3	240	112	128	Behavior	Yes	Increased anxiety and depression	Both	BASC-2
24	(73)	1.8	0.045	All	0.096	350	163	187	Behavior	No	NA	Both	NNNS
25	(74)	1.03	0.026	All	3	106	55	51	Skills	No	NA	Both	MSEL

MSEL, Mullen Scales of Early Learning; WISC, Weschler Intelligence Scale for Children; BASC, Behavioral Assessment System for Children; BSID, Bayley Scales of Infant Development; DSM-IV, Diagnostic and Statistical Manual of Mental Disorders 4th Edition; CBCL, Child Behavior Check List; SDQ, Strengths and Difficulties Questionnaire; CADS, Conner’s ADHD/DSM-IV Scales; MB-CDI, McArthur-Bates Communicative Development Inventories; SRS, Social Responsiveness Scale; GSD, Gesell Development Schedules; NCIU, Network Neurobehavioral Scale.

From these selected studies, 50% percent were done in the United States, 25% in Asia, 20% in Europe and 5% in Africa. Eighty percent of the studies (n = 16), associated BPA consumption with neurodevelopmental deficits including internalizing and externalizing behaviors, such as poor mental, social, adaptive and language development, and lower IQ.

The children neurodevelopment was evaluated at a different human growth and development period. One during infancy (< one year old), nine during early childhood (one- to three-year-olds), and 10 at the childhood. Two were between three and five years old, and eight were between six and ten years old.

#### 3.1.1 Infancy

During the first months of life, Yolton et al. (73) found no significant trends with respect to BPA levels (p>0.10). Samples were collected during the first and second trimester of pregnancy (N=350), with detectable concentrations of BPA in 90% of cases, and infants were given the NCIU Network Neurobehavioral Scale (NNNS) at 5 weeks after birth.

#### 3.1.2 Early childhood

Casas et al. (53), took urine samples from mothers (N=622) during the third trimester and detected BPA in 479 of them, with 382 undergoing neurodevelopmental testing in the first year. They found that BPA is related to a decrease in psychomotor development (β=-4.28, 95% CI: -8.15, -0.41, p<0.05) in one-year old children participating in the study “Childhood and the Environment of the city of Sabadell Spain” (INMA-Sabadell) using the Bayley Scales of Infant Development (BSID). In another study, Pan et al. (71) found BPA in the urine samples of 86.9% of the mothers (N=368) admitted to the hospital for delivery and assessed the potential relationship between BPA levels and neurodevelopment using the Gesell Development Schedules (GSD). The GSD test assesses four areas of neurodevelopment (motor, adaptive, language, and social) through a Development Coefficient score. An inverse relationship was found between BPA and total neurodevelopment with a 1.43-point decrease in the assessment each time the urine concentration increased 10-fold (β= -1.43, 95% CI: -2.30, -0.56, p=0.001) in both sexes.

In 2-year-old girls, Braun et al. (58) found a significant relationship between maternal urinary BPA concentration measured in the second and third trimesters (n= 249) and an increase in externalizing behaviors (β=6.0, 95% CI: 0.1, 12.0) in girls. They found that high BPA concentrations before 16 weeks were more associated with these behaviors in all children (β= 2.9, 95% CI: 0.2, 5.7), whereas the greatest effect prevailed in girls. Behavioral assessment was performed with the Behavioral Assessment System for Children (BASC-2).

Jensen et al. (66) Assessed BPA concentrations on average at week 28 in pregnant women (N=2217) belonging to the Odense Child Cohort (OCC). 796 participants had presence of the compound, of which 535 and 398 completed the vocabulary and complexity questionnaires, respectively. They compared it with language development between 18 and 36 months of age of children using the McArthur-Bates Communicative Development Inventories in its Danish adaptation (MC-CDI). They noted that high concentrations were associated with low language development in vocabulary (OR= 4.63 95% CI 1.74, 12.30) and complexity (OR= 2.43 95% CI 1.02, 5.77) in male children.

Jiang et al. (61) collected urine samples in each trimester of pregnancy from 856 women to obtain BPA concentrations and related the date to children’s neurodevelopment at 23–26 months of age using the BSID-I. They found a lower mental development at two years when BPA was observed in the second trimester (β= -2.87 95% CI: -4.98, -0.75; with a p value for trimester interaction= 0.04).

Kim et al. (68) took the urine sample of mothers (N=140) belonging to the Children’s Health and Environmental Chemicals in Korea (CHECK), of which the presence of BPA was detected in 85% when they were admitted for labor and assessed the neurodevelopment of children using the BSID-II. They found that a high concentration of BPA was related to low mental development only in girls (β= -4.07 95% CI: -7.61, -0.53, p<0.05). In turn, Pan et al. (71) found an inverse relationship between concentration and language development (β= -1.69, 95% CI: -3.23, -0.15, p = 0.032), (N=296) with the presence of BPA in 86.9% of the samples).

Braun et al. (72) analyzed urinary BPA concentrations in women in the second and third trimester of pregnancy (present in more than 97% of samples) using BASC-2. They found that all of them presented greater anxiety (β= 7, 95% CI: 1.7, 12, p<0.5) and depression (β= 4.9, 95% CI: 0.0, 9.9) at 3 years. In boys they observed a significant decrease in hyperactivity (β= -6.3, 95% CI: -12, -0.6) with respect to the values ​​of the BASC-2 study of a neurotypical person.

Zhou et al. (67) published a study using data from the South African Drakenstein Child Health Study, where they found that data of children (n=545) whose mothers urine BPA concentration was analyzed during the second trimester of pregnancy (1.95μg/l), showed a significant decrease on cognitive development in 2 years old males (β= -1.39; 95% CI: -2.54, -0.23) while there was no significant effect on girls (β= 0.56; 95% CI: -0.46, 1.56) based on BSID III test.

Barkoski et al. (74), conducted a study involving 207 mother-child pairs from the Markers of Autism Risk in Babies Learning Early Signs (MARBLES) cohort, where BPA was detected in 58.3% urine samples during the second and third trimester of pregnancy. A neurodevelopmental assessment of the children was performed when the children were already 3 years old using the Autism Diagnostic Observation Scale (ADOS) and the Mullen Scales of Early Learning (MSEL) and found no relationship between BPA concentration and autism spectrum disorder (ASD) while establishing that high concentrations of BPA during the second trimester have a borderline association with a low risk of non-neurotypical development (OR = 0.67, 95% CI: 0.43, 1.06).

However, in 2024 Oskar et al. (69) found a significant effect on girls cognitive and motor development (β= -0.63, 95% CI: -1-11, -0.15) according to MSEL test, whose mothers urine samples were collected during 3rd trimester of pregnancy and analyzed to obtain BPA concentration (0.8μg/l). Data used in this study was also from MARBLES cohort.

#### 3.1.3 Three-to-five-year period

Perera et al. (64) found a significant effect on neurodevelopment when assessing BPA concentrations in urine of 34-week pregnant women and when evaluating their children when they were between 3 and 5 years old using the Child Behavior Check List (CBCL); however, they found that BPA (present in more than 90% of samples) had a significant effect on behaviors such as reactivity (β= 1.62, 95% CI: 1.12, 2.32, p=0.008) and aggressive behavior (β= 1.29, 95% CI: 1.09, 1.53 p=0.003) in boys.

#### 3.1.4 Six-to-ten-year period

Another study examined urinary BPA concentrations of pregnant women (only women with BPA present in urine were included) at a mean of 27 weeks and its effects on the behavior of their offspring (n= 125) between 6 and 10 years (62). Behavioral assessment was performed with CBCL, and Diagnostic and Statistical Manual of Mental Disorders (DSM) IV scales found a relationship in somatic symptoms in boys (CBCL β= 0.28, p=0.03; DSM-IV β= 0.3, p=0.01) and an increase in shyness/depression, rule breaking, externalizing behaviors in addition to challenging behaviors and conduct disorder only in boys.

Conversely, Guo et al. (75) obtained urine samples (with a detection of BPA in 100% of the samples), from pregnant women (n=386) on the day of delivery using the Sheyang Mini Birth Cohort Study (SMBCS). Then, a neurodevelopment assessment using the Strengths and Difficulties Questionnaire (SDQ) of 10-year-old children found a significant relationship between BPA concentration and an elevated risk of total difficulties (emotional symptoms, conduct problems, prosocial behavior, hyperactivity/inattention) (OR = 1.57, 95% CI: 1.08, 2.28, P = 0.018) in boys.

Guo et al. (51), evaluated in the SMBCS the effect of prenatal BPA exposure and children’s IQ at age of 7 years using the Chinese version of the Weschler Intelligence Scale for Children (C-WISC). They found a significant relationship between the decrease in Full Intelligence Quotient (FIQ) of boys and the high concentration of BPA in their mothers (β=-1.18, 95% CI: -2.21, -0.15, p=0.025); with a p-value for the sex interaction = 0.296.

Harley et al. (60) took urine (with the presence of BPA in 100% of the samples) samples at approximately 13 and 26 weeks to subsequently assess children’s neurocognitive development at 7 years using the BASC-2 and Conner’s ADHD/DSM-IV Scales (CADS) and at 9 years old with the CADS alone. They found a significant relationship between BPA concentrations and internalizing behaviors in male children that increased (β= 1.8 95% CI: 0.3, 3.3, p<0.5) in the mother’s report and (β= 2.5 95% CI: 0.7, 4.4, p<0.01) in the teacher’s report, each time the BPA concentration doubled. In contrast, no Attention-Deficit/Hyperactivity Disorder (ADHD) symptoms were found.

The effect of BPA (present in 100% of samples) on the IQ of 7-year-old children from the WISC IV was evaluated by Tanner et al. (57) through the Swedish Environmental Longitudinal, Mother and child, Asthma and Allergy Study (SELMA) cohort. They found a decrease of the IQ only in male children (β= -1.9, 95% CI: -3.6, -0.2, p<0.05) who were exposed to a mixture of endocrine disruptors where BPF was associated 14% and BPA 4% with a negative effect.

Roen et al. (65) obtained the sample during the third semester (with the presence of BPA in 98% of the samples) and compared it with behavior in 7 - 9-year-old children using CBCL and found that girls presented lower internalizing (β= -0.17, p=0.04) while boys presented higher internalizing (β= 0.41, p<0.0001) and externalizing (β= 0.40, p<0.0001) symptoms as BPA concentration increased.

Miodovnik et al. (70) assessed BPA concentrations, present in 90% of the samples, in pregnant women (N=407) during the third trimester and ASD-related social disability of their children between 7 and 9 years old (N=137) using the Social Responsiveness Scale (SRS). No significant association was observed (β= 1.18, 95% CI -0.75, 3.11, p>0.05). Furthermore, Bornehag et al. (59) found no significant relationship between IQ and BPA (present in 90% of samples) (β= -0.51 95% CI: -3.14, 2.13 P = 0.705) in the SELMA cohort study using the WISC IV test.

### 3.2 Risk for infant neurodevelopment

The estimated daily intake of Bisphenol A at which a negative effect on neurodevelopment and neurobehavior was observed between 0.01 and 0.08μg/kg body weight per day. On average, the studies show an adverse effect with intake values ​​that are below the safe levels set forth by international institutions.

A sex-dependent effect was found. Among girls, there was an increase in externalizing behaviors (58), a decrease in internalizing behaviors (65), and a deficit in mental development (68, 69). In boys, there was an increase in externalizing and internalizing behaviors (53, 60, 62, 63, 65, 76)decreased IQ (51, 57), and low mental (61, 67), verbal (66) and social (71) development.

According to the articles consulted, the deficit was more likely to be identified when high concentrations of BPA were detected in the third trimester of gestation (61% of the results). Biologically, the earliest the exposure to contaminants during the development of the gestational product, the greatest negative impact is expected. This finding should be further researched and confirmed through more clinical studies. At doses lower than those established by international authorities, the authors that evaluated a sex dependent effect, found that it is more probable for males (65% of the results) to show affectation associated with the brain.

BPA’s endocrine disruption is mediated by activation of ERRγ and GPR30 eliciting a diverse range of physiological effects. For example, within adipose tissue, BPA-GPR30 signaling augments pro-inflammatory cytokine levels, modifies adipokine expression, and promotes cell proliferation, potentially contributing to the development of obesity and metabolic syndrome (77, 78). In reproductive tissues, BPA’s binding to GPR30 induces apoptosis in spermatocytes and disrupts both placental and testicular function, thereby implicating it in infertility and developmental abnormalities (78–80). In various cancer models, BPA promotes the proliferation of breast, thyroid, and testicular tumor cells via ERRγ and GPR30 (80–83).

At a molecular and cellular level, BPA induces oxidative stress, inflammation, apoptosis, and disruptions in key signaling pathways such as Wnt, PI3K/Akt, and MAPK (83). Furthermore, it can modify Deoxyribonucleic Acid (DNA) methylation, histone structure, and micro Ribonucleic acid (RNA) expression, contributing to persistent epigenetic and transgenerational effects (84). The disruption of redox homeostasis and the alteration of mitochondrial function also represent crucial mechanisms in BPA’s toxicity (85). Additionally, BPA exposure compromises immune cell function by inhibiting telomerase activity, inducing DNA damage, and reducing proliferation in T cells and peripheral blood mononuclear cells (PBMCs), primarily through ER/GPR30-ERK signaling. These effects are observed at low, environmentally relevant concentrations and are reversible upon the removal of BPA (86).

Numerous studies demonstrate that BPA alters DNA methylation, histone modification, and microRNA expression, thereby impacting key genes essential for proper brain development. These epigenetic modifications can be heritable and contribute to enduring changes in neuronal function and behavior, including an increased susceptibility to neuropsychiatric disorders (87). BPA functions as an agonist/antagonist of both estrogenic and androgenic receptors, thereby interfering with the hormonal signaling pathways critical for neuronal differentiation and maturation (88). This disruption can consequently alter neurogenesis, neuronal migration, and the formation of cerebral circuits, with potential effects that are both sex-specific and transgenerational (89). BPA adversely affects the proliferation and differentiation of neural stem cells, the formation and maturation of synapses, and the overall morphology of neurons and glia, particularly within crucial regions such as the hippocampus and cerebral cortex (90). Observable outcomes include a reduction in neurogenesis, alterations in neuronal migration, decreased spine density, and changes in synaptic plasticity (91). It also affects the expression of vital neurotrophic factors, such as brain-derived neurotrophic factor (BDNF) and insulin-like growth factor 1 (IGF-1), and perturbs the homeostasis of key neurotransmitters like gamma-aminobutyric acid (GABA), glutamate, dopamine, and monoamines (92–96). Additionally, BPA can induce oxidative stress, neuroinflammation, and mitochondrial dysfunction, all contributing to its neurotoxic effects (9).

The initiation of neurodevelopment takes place during gestation, involving the maturation of the nervous system between the 3rd and 4th weeks of pregnancy and the development of the brain from the 9th week onward. These processes are closely linked to the regulation of body coordination and behavior. A network of precursor cells of neurons is generated during pregnancy, where the interconnectivity is of vital importance for neurodevelopment after birth (97). For example, the hippocampus regulates recognition and spatial memory, and both these cognitive processes undergo major developmental changes between 32 weeks of pregnancy and 18 months of age. Similarly, the prefrontal cortex, which is the region regulating executive functions, such as attention and multitasking, reaches a developmental milestone at around 6 months of age. Finally, the histaminergic, catecholaminergic (dopamine, epinephrine, and norepinephrine), and serotonergic signaling neurotransmitter systems develop from pregnancy to 3 years of age and regulate the reward pathway, mood, and affection (98).

Estrin and Bhavnani (99) have reported that the gray matter of the fetus increased 10 times from week 29 to the day of delivery, together with several brain connections leading to the fact that brain maturation occurs mainly during the 3rd trimester. This agrees with our findings where BPA exposure during this period shows the highest neurodevelopment risk for children.

Mhaouty-Kodja et al. (100) conducted a review on neurodevelopment and BPA in animals, noting that males exhibited more significant impacts on spatial memory following exposure compared to females. Similarly, our findings indicate that males face a higher risk of deficits in neurodevelopmental memory.

It’s crucial to emphasize that the concept of “safe levels of intake” for BPA is somewhat arbitrary, given the biological variability among individuals, making some more susceptible to its deleterious effects than others. International food safety agencies typically estimate safe intake levels of environmental contaminants through diet, relying on parameters such as the no-observed-effect-level (NOEL) and the no-observed-adverse-effect-level (NOAEL). The NOEL is defined as the dose at which no detectable effects occur in an organism under specified experimental conditions (101).

In addition, according to the exposome theory, exposure to BPA can be added to exposure to other EDCs. The exposome encompasses all environmental exposures from conception to death, such as, chemical, physical, biological, and social, integrating both external and internal exposures (102). Its application in evaluating endocrine disruptors allows for the analysis of multiple and chronic exposures, as well as the effects of mixtures. This is essential for understanding the actual impact of compounds such as BPA and its substitutes (103). BPA substitutes, such as Bisphenol S (BPS), Bisphenol F (BPF), and Bisphenol AF (BPAF), have demonstrated in *in vitro*, *in vivo*, and epidemiological studies, similar or even superior hormonal potencies to BPA, affecting processes such as adipogenesis, cell differentiation, thyroid function, and neurodevelopment (104, 105). The exposome approach has revealed that simultaneous exposure to BPA and its analogs can lead to additive or synergistic effects, particularly in the activation or inhibition of hormone receptors and in the alteration of effect biomarkers (e.g., sex hormones, oxidative stress markers, inflammation). Recent studies indicate that mixtures of bisphenols can induce endocrine effects at lower concentrations than individual compounds (106). Despite these advances, significant challenges persist. These include the precise measurement of exposures, the interpretation of low-dose effects, the evaluation of mixtures, and the extrapolation of animal model results to humans. Furthermore, there are gaps in the toxicological characterization of many BPA substitutes and in the identification of specific effect biomarkers (107).

### 3.3 Effect’s magnitude analysis

A variety of tests, including BASC-2, BSID, CBCL, and WISC, were extensively employed in the studies under review, with the majority indicating a notable impact on neurodevelopment.

**Figure 6:**
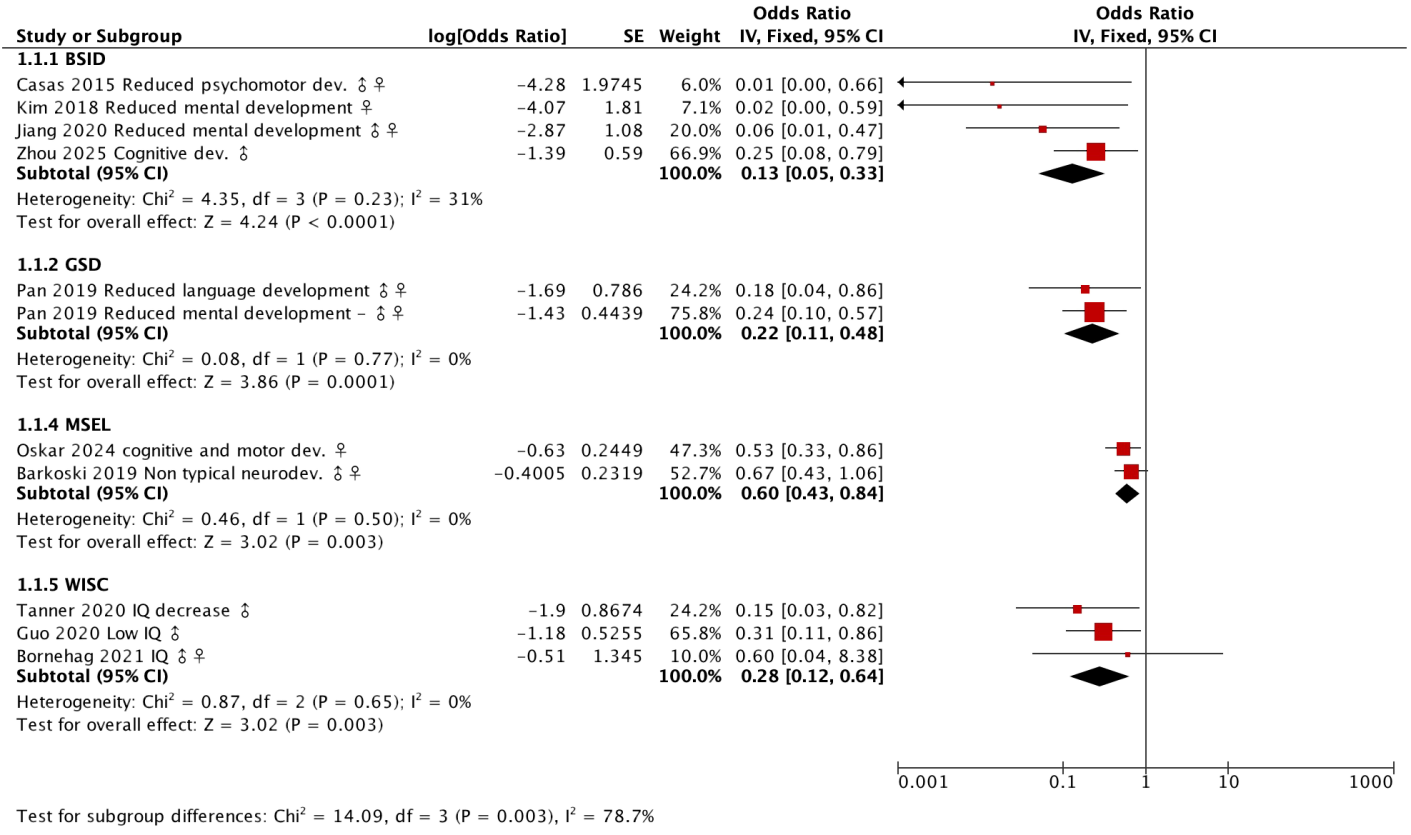
Magnitudes of the effect of BPA on cognitive abilities in neurodevelopment. The effects reported in terms of the odds ratio for each of the tests used in the studies were compared and it was found that the BSID test obtains more conclusive results.

**Figure 7:**
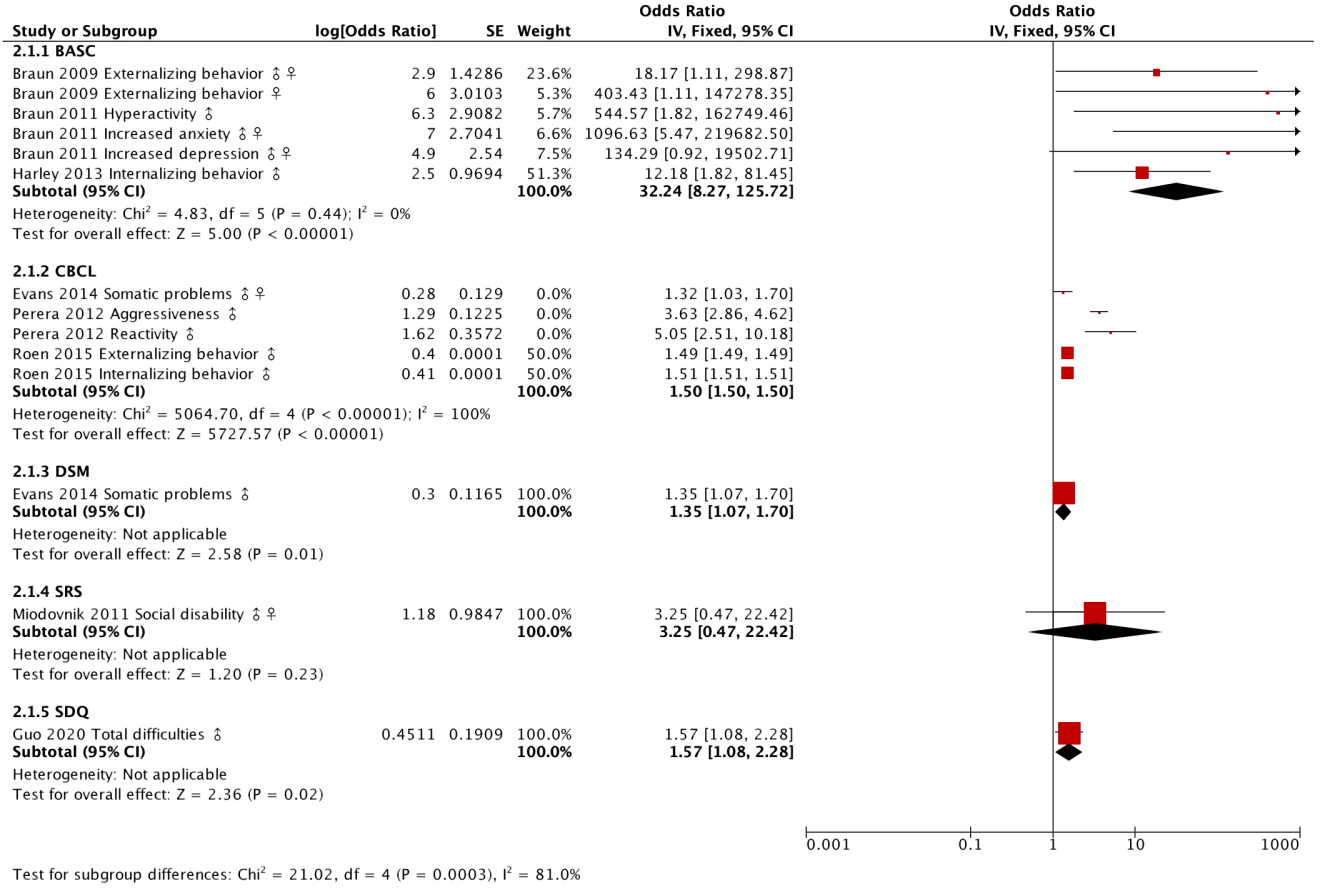
Magnitudes of the effect of BPA on behavior in neurodevelopment. A comparison of the odds ratio of each test reported in the reviewed studies is shown and it was found that the BASC test obtains more conclusive results.

**Figure 7** shows the magnitudes of the effect of BPA on cognitive abilities in neurodevelopment. We can see that the BSID evaluation is the one that obtains the greatest effect of the included studies when evaluating a deficit in the mental and psychomotor development of infants. On the other hand, the GSD, WISC and MC-CDI evaluations show significant effects on language development, IQ, and mental development. The graph shows the statistical weight of each result when estimating the sample size by means of the standard error, therefore, although Bornehag et al. (59) did not find significance in his study, its statistical weight is less than that of the results of Guo et al (51) and Tanner et al. (57), who performed the same evaluation and obtained the opposite. The diamonds situated within the intervals of each test depict the average effect of that evaluation. Interestingly, even studies yielding non-significant results, the average effect across all evaluations remains significant.

Looking into behavioral evaluations in **Figure 7**, the BASC shows the greatest effect, scrutinizing both internalizing and externalizing behaviors. It’s crucial to note that while Braun contributes significantly to this category, having the highest number of published papers and large effects, the statistical weight of his results is relatively low compared to other authors. Hence, the significance of the average effect in this test cannot be solely attributed to Braun’s contributions.

Despite some weak effects observed in assessments by Roen et al. (65) and the unreported non-significant effects by Evans et al. (62), the cumulative average effect across all tests reveals a statistically significant impact on behavior, except for the SRS, exclusively used by Miodovnik et al. (70).

Further, it’s important to clarify that the negative effects identified by Braun et al. (72) and Roen et al. (65) indicate a decrease in certain behaviors compared to the average and aren’t tied to the statistical significance of the result.

To sum up, 16% of the studies didn’t found a significative relation between BPA exposure and neurodevelopment. Specifically (74) and (59) found no significant relation when analizing the same SELMA cohort study data that other authors revised.

Finally, it is important to consider that many neurodevelopmental consequences of unintended exposure to BPA or other emerging contaminants in pregnant mothers are clearly observable until the child’s school age. This means a period of at least 4 years in which there could be a therapeutic intervention to maximize the integral health in the neurodevelopment of an infant and improve its perspective of quality of life in adulthood. In this regard, it is worth assessing whether the neurodegenerative disorders associated with advanced age could have their origin in infant neurodevelopment.

The quality of life of neurodivergent individuals depends significantly on their ability to interact with their environment and their ability to read and write (108). This could lead to the importance of developing prenatal screens for the collection of information and design of strategies to mitigate neurodevelopmental disorders during early childhood (0 to 12 years).

## 4 Limitations

Cross-sectional studies simultaneously assess BPA exposure and health outcomes, which limit their interpretability, especially for results that have long latency periods. Given the short half-life of BPA, the use of a single urine sample to categorize exposure is another limitation in most human studies. BPA data was based on urine concentration only. Use of urine is a non-invasive determination method which might be best suitable towards a public health policy for the mother-child pair. Furthermore, no BPA metabolites were considered in our analysis [eg. BPA glucoronide, sulfonated BPA and more recelty MBP-4-Methyl-2,4-bis (4-hydroxyphenil) pent-1-ene].

## 5 Conclusions

We performed a comprehensive assessment of available literature on prenatal exposure to bisphenol A as a risk factor for childhood neurodevelopment. Evidence collected from various authors at different geographic locations indicates a potential risk towards neurodevelopmental deficits for male offspring in women ingesting at least 0.01μg/kg bw/day of BPA, particularly during the third trimester of pregnancy.

We also found that the tests that provide a greater magnitude of effect, and therefore a more conclusive result, are the Behavioral Assessment System for Children (BASC) for behavior and the Bayley Scales of Infant Development (BSID) for cognitive abilities.

The maximum doses of BPA established by FDA, are above the doses at which neurodevelopmental effects of BPA were found in this study. Consequently, we consider that international regulations should be reassessed considering these scientific findings to further quantify the prevalence and incidence of BPA and its association with neurodevelopmental disorders for the design of new public health policies.

Finally, negative neurodevelopmental outcome in children has been reported from 0.01 to 0.08μg/kg bw/day. Recent EFSA proposal to reduce t-TDI from 4μg/kg bw/day to 0.00002μg/kg bw/day could be adequate to protect neurodevelopmental deficits due to prenatal exposure to BPA. However, the non-monotonic behavior of BPA, still in debate, rethinks the use of resources such as the NOAEL and the TDI to generate strategies that protect the population from adverse health effects, at least due to BPA.

## Glossary

MBP: 4-Methyl-2,4-bis(4-hydroxyphenyl)pent-1-ene
ADHD: Attention-deficit/hyperactivity disorder
ADOS: Autism Diagnostic Observation Scale
ASD: autism spectrum disorder
W: average weight
BSID: Bayley Scales of Infant Development
BASC: Behavioral Assessment System for Children
BPA: Bisphenol A
BPA-G: Bisphenol A glucoronide
BPAF: Bisphenol AF
BPF: Bisphenol F
BPS: Bisphenol S
BDNF: brain-derived neurotrophic factor
CBCL: Child Behavior Check List
INMA-Sabadell: Childhood and the Environment of the city of Sabadell Spain
CHECK: Children&rsquo;s Health and Environmental Chemicals in Korea
CADS: Conner&rsquo;s ADHD/DSM-IV Scale
DI: daily intake
DNA: Deoxyribonucleic Acid
DOHaD: Developmental Origins of Health and Disease
DSM: Diagnostic and Statistical Manual of Mental Disorders
EDC: endocrine disrupting compound
ER: estrogen responsive
EDs: endocrine disruptors
EFSA: European Food Safety Authority
FDA: Food and Drug Administration
GABA: gamma-aminobutyric acid
GSD: Gessel Development Schedules
IGF-1: insulin-like growth factor 1
IQ: intelligence quotient
MARBLES: Markers of Autism Risk in Babies Learning Early Signs
MC-CDI: Mc Arthur-Bates Communicative Development
MSEL: Mullen Scales of Early Learning
NNNS: NCIU Network Neurobehavioural Scale
NOAEL: non-observed-adverse- effect-level
NOEL: non-observed-effect-level
OR: odds ratio
OCC: Odense Child Cohort
PBMCs: peripheral blood mononuclear cells
RfD: Reference dose
RNA: Ribonucleic acid
SMBCS: Sheyang Mini Birth Cohort Study
SRS: Social Responsiveness Scale
SDQ: Strengths and Difficulties Questionnaire
SELMA: Swedish Environmental Longitudinal, Mother and child, Asthma and Allergy Study
t-TDI: temporary tolerable daily intake
TDI: tolerable daily intake
FIQ: total intelligence quotient
EPA: United States Environmental Protection Agency
UDP: uridine diphosphate
UC: urine concentration
UV: urine volume
WISC: Weschler Intelligence Scale for Children
